# Decreasing ventromedial prefrontal cortex deactivation in risky decision making after simulated microgravity: effects of −6° head-down tilt bed rest

**DOI:** 10.3389/fnbeh.2014.00187

**Published:** 2014-05-27

**Authors:** Li-Lin Rao, Yuan Zhou, Zhu-Yuan Liang, Henyi Rao, Rui Zheng, Yan Sun, Cheng Tan, Yi Xiao, Zhi-Qiang Tian, Xiao-Ping Chen, Chun-Hui Wang, Yan-Qiang Bai, Shan-Guang Chen, Shu Li

**Affiliations:** ^1^Key Laboratory of Behavioral Science, Magnetic Resonance Imaging Research Center, Institute of Psychology, Chinese Academy of SciencesBeijing, China; ^2^Department of Psychology, Sun Yat-Sen UniversityGuangzhou, China; ^3^China Astronaut Research and Training CenterBeijing, China

**Keywords:** risk, BART, fMRI, bed rest, ventromedial prefrontal cortex

## Abstract

Space is characterized by risk and uncertainty. As humans play an important role in long-duration space missions, the ability to make risky decisions effectively is important for astronauts who spend extended time periods in space. The present study used the Balloon Analog Risk Task to conduct both behavioral and fMRI experiments to evaluate the effects of simulated microgravity on individuals' risk-taking behavior and the neural basis of the effect. The results showed that participants' risk-taking behavior was not affected by bed rest. However, we found that the ventromedial prefrontal cortex (VMPFC) showed less deactivation after bed rest and that the VMPFC activation in the active choice condition showed no significant difference between the win outcome and the loss outcome after bed rest, although its activation was significantly greater in the win outcome than in the loss outcome before bed rest. These results suggested that the participants showed a decreased level of value calculation after the bed rest. Our findings can contribute to a better understanding of the effect of microgravity on individual higher-level cognitive functioning.

## Introduction

Space is characterized by risk and uncertainty. Humans have begun to study issues concerning interplanetary spaceflight (Basner et al., 2013). Long-duration space missions pose unique challenges and unexpected risks, and a successful space mission requires effective risk management. Because humans play an important role in long-duration space missions, the ability to make risky decisions effectively is important for astronauts who spend extended periods of time in space. Surprisingly, few studies have investigated astronauts' risk-taking behavior (De la Torre et al., 2012). The current study was the first attempt to use both behavioral and fMRI experiments to evaluate the effect of simulated microgravity on individuals' risk-taking behavior and the neural basis of this effect.

Although the state of weightlessness as experienced by astronauts during space missions is difficult to recreate on Earth, head-down tilt bed rest (i.e., prolonged periods of rest in the lying-down position) has proved its usefulness as a reliable simulation model for most physiological effects of spaceflight (Pavy-Le Traon et al., 2007; Nicolas and Weiss, 2009; Moore et al., 2010). Researchers previously focused on physiological changes after bed rest, such as bone mineral and lean tissue loss (LeBlanc et al., 1992). Currently, researchers have directed increasing attention to psychological issues. For example, numerous studies have investigated whether cognitive functions are affected during bed rest (Shehab et al., 1998; Dolenc et al., 2008; Lipnicki and Gunga, 2009; Lipnicki et al., 2009a; Seaton et al., 2009a,b; Jiang et al., 2013). Evidence for a detrimental effect of bed rest on executive functioning has been reported (Lipnicki et al., 2009a). In contrast, evidence for an improvement in cognitive function following bed rest has also been found (DeRoshia and Greenleef, 1993). Lipnicki and Gunga (2009) reviewed 17 bed rest studies and found that the reported effects of bed rest on cognitive performance vary considerably, from worsening (as generally expected) to improvement.

However, far less is known about the possible effects of simulated microgravity on individuals' risk-taking behavior. As a high-level cognitive function, risky decision making plays an important role in the activities of individuals in an uncertain and isolated environment during exposure to weightlessness or simulated weightlessness. Bed rest studies involve limited social contacts between participants and the exterior environment. Lack of social contacts and isolation from a familiar environment might contribute in some way to the psychological and behavioral changes occurring during a period of prolonged bed rest (Dolenc et al., 2008). Previous studies have shown that higher levels of loneliness or social isolation might be associated with increases in risk seeking (Trevorrow and Moore, 1998). On the other hand, the participants might have more depressive symptoms and a poorer mood status during bed rest (Styf et al., 2001; Ishizaki et al., 2002). Studies using depressive individuals as participants have suggested enhanced feedback-based decision making and risk aversion among depressive individuals (Smoski et al., 2008). Lipnicki et al. (2009a,b) found that participants' scores on the Iowa Gambling Task were significantly worse during bed rest than during ambulatory sessions, but they did not test the effect of bed rest on risk preference. Thus, no specific hypothesis was formulated regarding differences in risk-taking behavior during bed rest.

The Balloon Analog Risk Task (BART) is considered to be an ecologically valid method of measuring individuals' risk-taking behavior (Lejuez et al., 2002). In this task, the participants are asked to sequentially inflate (pump) a balloon that can either grow larger or explode. As the risk is ecologically defined as the probability of explosion for each balloon in the BART, neuroimaging researchers have used this task to identify brain regions related to risk, including the dorsal lateral prefrontal cortex, anterior cingulate/medial frontal cortex, ventral and dorsal striatum, anterior insula, and midbrain (Rao et al., 2008; Bogg et al., 2012; Cazzell et al., 2012; Chiu et al., 2012; Lighthall et al., 2012; Schonberg et al., 2012; Telzer et al., 2013). Specifically, Schonberg et al. (2012) have noted that the ventromedial prefrontal cortex (VMPFC) plays an important role in the BART. They found that VMPFC activation decreased as the participants further expanded the balloons, suggesting that the participants might focus their attention on the potential losses from each additional pump rather than on the sequential marginally added value. In addition, because feedback (i.e., win or loss) is provided in the BART task, this design enables neuroimaging researchers to investigate brain activation that is sensitive to feedback outcomes. Previous studies have identified brain regions related to outcomes, including the VMPFC, striatum, insula, and anterior cingulate cortex (Rao et al., 2008; Schonberg et al., 2012).

Thus, in the current study, by integrating behavioral and fMRI experiments, we used the BART to examine whether a 45-day period of head-down tilt bed rest would influence individuals' risk-taking behavior and to explore the neural basis of this effect.

## Materials and methods

### Sample

Sixteen healthy adult males aged between 20 and 34 (*M ± SD*, 26.6 ± 4.2), participated in the study. The selection process involved two steps: the first step was an interview about the past history and present condition of the participant in terms of physical and psychosocial status, and the second step included a physical examination in which routine medical and laboratory analyses were used to exclude chronic diseases. The 16 participants selected had education above the high school level and took no medications or drugs.

All participants were in good health with no previous history of psychiatric or neurological disease. Written informed consent was obtained following a detailed explanation of the study (purpose and research hypotheses, experimental procedures and methods, research conditions, possible problems, and complications). The participants were given a financial award at the end of the study. One participant was excluded from the fMRI analyses because of excessive head motion (see fMRI Data Preprocessing section). The study was approved by the Institutional Review Board of the Institute of Psychology, the Chinese Academy of Sciences and the Institutional Review Board of the China Astronaut Research and Training Center.

### Procedure

The experiment consisted of three stages: a 10-day baseline control period (pre-BR), a 45-day head-down tilt bed rest period (BR), and a 10-day post-BR ambulatory recovery period (post-BR).

The participants remained ambulatory for the first 10 days of the study. During this period, they acclimated physiologically and psychologically to the bed rest facility, the study diet regimen, and the regulation of circadian cycles. Two pre-bed rest tests, one in 4 days before bed rest for the behavioral experiment and the other in 3 days before bed rest for the fMRI experiment, were scheduled for each participant during this phase to establish a baseline for the participants' subsequent behavioral and neuroimaging BART performance.

The participants began the 45-day bed rest phase on the 11th day of the study. They were confined to a −6° head-down bed rest for 24 h/day during the 45-day bed rest phase, and completed one session of behavioral test once a week for a total of six behavioral tests. The participants were housed in three rooms and were under constant video surveillance. They performed all daily activities lying down and were not permitted to leave their beds. The bedrooms were air-conditioned, and the room temperature was maintained between 23 and 27°C. Nursing care was provided throughout the duration of the study. Physicians regularly checked the physical condition of the participants. The participants were allowed to communicate freely with each other, to watch television and videos, to listen to radio and tapes, to read books and magazines, and to make calls. The equipment was positioned on the floor using an apparatus to elevate and appropriately tilt the Thinkpad device.

Following the bed rest phase, the participants began a 10-day recovery phase during which they remained in the bed rest facility but returned to ambulation. Each participant completed two post-bed rest tests beginning on the third day of return to ambulation (for the fMRI experiment) and 4 days thereafter (the seventh day of return to ambulation for the behavioral experiment).

In total, the participants performed two fMRI sessions (pre- and post-) and eight behavioral sessions.

### Task and experimental design

Two versions of the BART were used to measure participants' risk-taking behavior, one for the behavioral experiment and the other for the fMRI experiment.

#### Behavioral version of the BART

**Figure 1:**
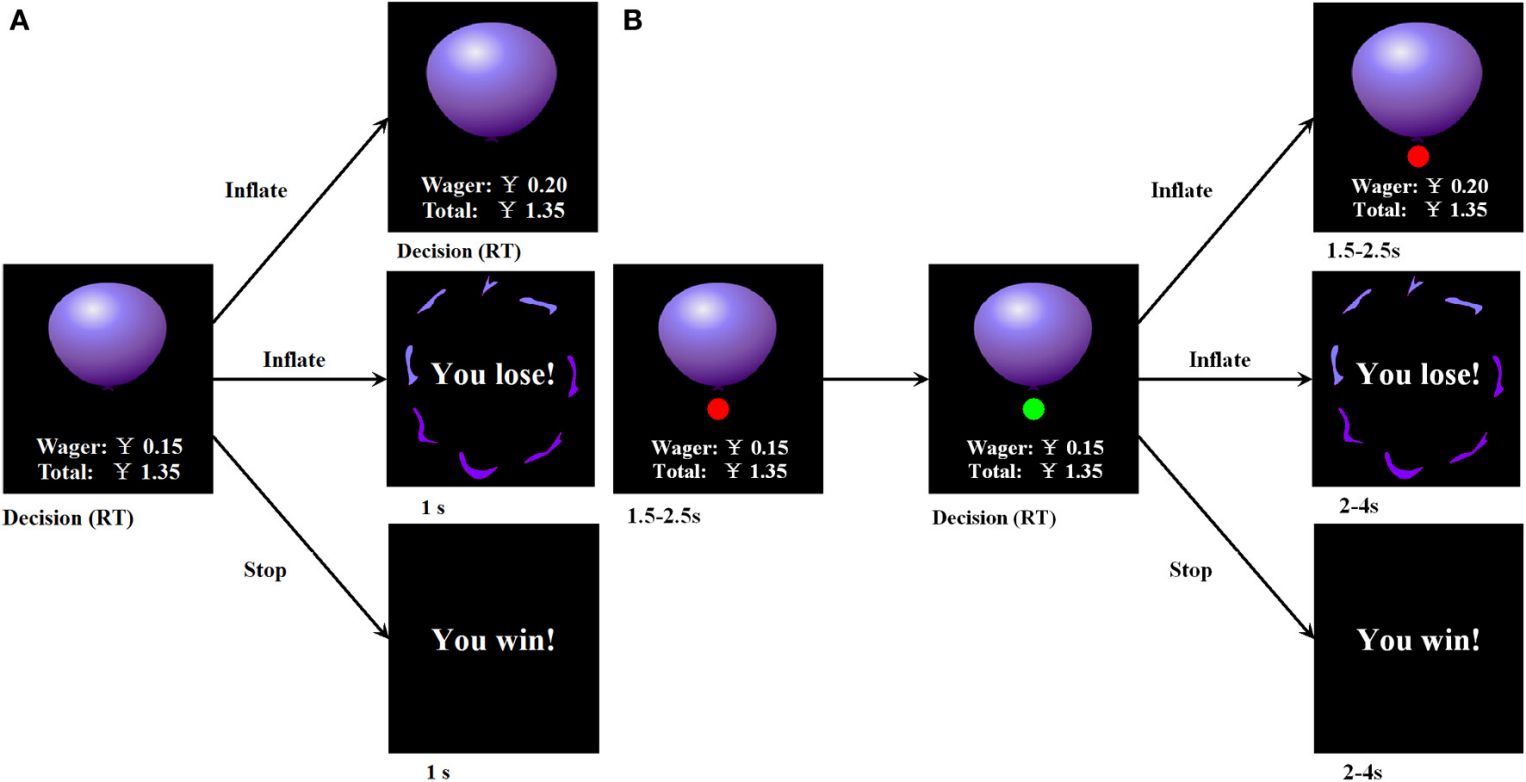
**Overview of (A) behavioral and (B) fMRI-adapted versions of the BART procedure and presentation**.

The behavioral version of the BART was adapted from Lejuez et al. (2002). The participants were presented a virtual balloon and asked to press one of two buttons to either inflate (pump) the balloon or to cash out (Figure 1). As the balloon was inflated, both the monetary reward and the probability of explosion increased. Participants received a reward (50 Chinese cents) for each pump on which the balloon was successfully inflated. They could stop inflating the balloon at any point and keep the accumulated reward. The maximum number of pumps that participants could deliver to each balloon was 128. The probability that a balloon would explode was arranged by constructing an array of 128 numbers. The number 1 was assigned to indicate a balloon explosion. On each pump of the balloon, a number was selected without replacement from the array. The balloon exploded if the number 1 was selected. If the balloon exploded, the participants would lose the money accumulated on that balloon trial alone (a balloon trial began with the presentation of a balloon and ended either when the balloon exploded or the participant cashed out). Each participant completed 30 balloon trials. The maximum number of inflations and the exact probability of explosion were unknown to the participants.

#### fMRI-adapted version of the BART

The fMRI-adapted version of the BART used in this study (Figure 1) was guided by prior imaging work (Rao et al., 2008). The fMRI-adapted BART was generally similar to the behavioral BART but also involved several differences. The maximum number of pumps that participants could make for each balloon was 12. The probability that a balloon would explode was arranged by constructing an array of 12 numbers. The timing of the inflation was controlled by a cue, which consisted of a small circle that changed color from red to green with a jittered time interval (Figure 1). The participants could press a button to continue or discontinue inflation only when the color of the cue was green. After the participants successfully pressed a button and inflated the balloon, the cue immediately turned red for a random interval between 1.5 and 2.5 s. The cue then turned green again to indicate the next inflation. After the end of each balloon trial, there was also a jittered 2–4 s interval prior to the presentation of the next balloon.

The fMRI-adapted BART included an active choice condition and a passive no-choice condition. In the active choice condition, the participants were asked to decide either to inflate the balloon or to cash out. In the passive no-choice condition, the participants were asked to inflate the balloon continually, and the computer determined the end point as well as the win or loss outcomes for each balloon. The number of balloons that participants completed during the scan was not pre-determined in either the active or the passive choice conditions. Instead, the number depended on the response speed, which varied among the participants. Each participant completed a 6-min BOLD scan for the active choice condition and a 6-min BOLD scan for the passive choice condition. Prior to entering the scanner, the participants played a practice version to minimize learning effects during the actual scanning and to enable them to fully understand the paradigm.

### Behavioral analysis

In both the behavioral and fMRI experiments, we calculated the total and average number of pumps, the total number of wins (cash-out trials) and losses, the total and average number of pumps only for trials when the participant cashed out before the balloon exploded (hereafter termed “adjusted pumps”), the average adjusted pumps following a win trial, the average adjusted pumps following a loss trial, and the average reaction time (RT) for all pumps. We performed repeated-measures ANCOVAs with age as a covariate to compare these variables across choice modes and bed rest sessions. Statistical analyses of behavioral data were conducted using SPSS 17.0.

### fMRI data acquisition

The fMRI data in this article were acquired in the BNU Imaging Center for Brain Research, National Key Laboratory of Cognitive Neuroscience, and Learning. MR images sensitized to changes in BOLD signal levels were obtained by an echo planar imaging sequence on a 3.0-Tesla Siemens MR scanner (repetition time = 2000 ms; echo time = 30 ms; flip angle = 90°, matrix = 64 × 64; field of view = 200 × 200 mm^2^; slice thickness = 3.5 mm; slice gap = 0.7 mm). Each brain volume was composed of 33 axial slices. Stimuli were presented with E-prime software (Psychology Software Tools, Pittsburgh, PA, USA) on a personal computer, back-projected onto a screen using a liquid crystal display projector and viewed by the participants through a mirror mounted on the MRI head coil. The scanner was triggered by a signal generated by E-prime stimulus presentation software to synchronize each volume acquisition with the onset of a visual stimulus.

### fMRI data preprocessing

Data preprocessing and analyses were conducted with SPM8 software (Wellcome Department of Cognitive Neurology, University College London, London, UK, http://www.fil.ion.ucl.ac.uk/spm) running under MATLAB 7.10 (The MathWorks, Inc, Natick, Massachusetts, USA). Functional images were slice time-corrected to the onset of the middle slice and spatially realigned using a six-parameter affine transformation. Based on a visual inspection of the motion correction estimates, one participant who had more than 3-mm maximum displacement in any of the x, y, or z directions or more than 3° of angular rotation about any axis was excluded from this study. The realigned images were spatially normalized to the standard EPI template, resampled to 3 × 3 × 3 mm and subsequently smoothed with a Gaussian kernel of 8 mm full-width at half-maximum. Motion parameters were stored and used as nuisance variables in the following analysis.

Individual data were analyzed using a general linear model (GLM). Events were modeled with a variable-duration boxcar function convolved with a canonical hemodynamic response function (HRF). Participant-specific movement parameters were modeled as covariates of no interest. A high-pass filter with a cutoff period of 128 s was used to remove low-frequency noise.

### fMRI data analyses and statistics

We used event-related analyses to examine the differences in brain activation between the pre-bed rest and the post-bed rest. For each participant, two GLMs were conducted: one for the pre-bed rest test session and the other for the post-bed rest test session. Each GLM included six main events which resulted from a button press: an inflation of the balloon (i.e., a larger balloon) either in the active or passive choice conditions, a win outcome either in the active or passive choice conditions, or a loss outcome either in the active or passive choice conditions. The risk level associated with each inflation (i.e., the probability of explosion) was also entered into the model as a linear parametric modulation of the balloon inflation regressor. In the first level analyses, the following contrasts were computed for each participant: a contrast of risk (pre-bed active risk, post-bed active risk, pre-bed passive risk, and post-bed passive risk), a contrast of win outcome (pre-bed active win, post-bed active win, pre-bed passive win, and post-bed passive win), and a contrast of loss outcome (pre-bed active loss, post-bed active loss, pre-bed passive loss, and post-bed passive loss).

Second-level random effect analyses were completed by performing a 2 (time: pre- or post-bed) × 2 (choice condition: active or passive) ANOVA on the risk-related contrasts and a 2 (time: pre-bed or post-bed) × 2 (choice condition: active or passive) × 2 (outcome: win or loss) ANOVA on the outcome-related contrasts, with age as a covariate. A *post-hoc t*-test was conducted to examine the significance and direction of any effect. To control for Type I error, Monte Carlo simulations were performed (parameters were: individual voxel *p*-value = 0.001, 1,000 simulations, estimated FWHM using the statistical map, cluster connection radius *r* = 5 mm, with a gray mask with 55,342 voxels) using the AlphaSim program in the REST 1.8 software (http://www.restfmri.net). According to the simulations, a corrected significance level of *p* < 0.01 could be obtained with individual voxel height threshold of *p* < 0.001 and a cluster size dependent on the simulation to each statistical map.

## Results

### Behavioral experiment

Table 1 presents the results of the behavioral experiment. A repeated-measures One-Way ANCOVA with age as the covariate showed that there was no significant effect of time on the total number of pumps, *F*_(1, 7)_ = 0.419, *p* = 0.888; the average number of pumps, *F*_(1, 7)_ = 0.415, *p* = 0.891; the total number of wins, *F*_(1, 7)_ = 0.516, *p* = 0.821; the total number of losses, *F*_(1, 7)_ = 0.516, *p* = 0.821; the total earnings, *F*_(1, 7)_ = 1.154, *p* = 0.336; the average number of adjusted pumps, *F*_(1, 7)_ = 0.446, *p* = 0.870; the total number of adjusted pumps, *F*_(1, 7)_ = 0.449, *p* = 0.846; or the reaction time, *F*_(1, 7)_ = 0.498, *p* = 0.834. A 2 (previous feedback: win or loss) by 8 (time) repeated-measures ANCOVA with age as the covariate conducted on the average adjusted pumps following a win or a loss also revealed no significant effects of time, *F*_(7, 98)_ = 0.467, *p* = 0.856; feedback, *F*_(1, 14)_ = 3.145, *p* = 0.098; or interaction, *F*_(7, 98)_ = 1.396, *p* = 0.216. These results indicated that the participants' risk-taking behavior did not change significantly following the bed rest period.

**Table 1:** **The results of the behavioral experiment**.

		**1**	**2**	**3**	**4**	**5**	**6**	**7**	**8**
Pumps	*M*	36.86	36.15	35.68	36.72	35.77	36.66	38.94	36.95
	*SD*	14.95	8.80	5.59	6.36	5.37	4.22	7.25	3.28
Total of pumps	*M*	1105.88	1082.88	1070.25	1101.50	1073.13	1099.81	1168.31	1108.56
	*SD*	448.60	266.08	167.83	190.78	161.24	126.74	217.46	98.30
Adjusted pumps	*M*	43.49	42.46	43.39	43.39	42.12	42.04	44.53	43.09
	*SD*	21.29	13.96	10.14	8.23	8.61	4.04	7.06	5.65
Total of adjusted pumps	*M*	763.81	825.25	821.19	860.06	823.44	859.94	824.13	842.38
	*SD*	183.96	121.06	100.71	138.70	94.21	106.56	199.06	101.34
Win trials	*M*	20.25	20.63	19.50	20.25	20.19	20.50	18.56	19.75
	*SD*	5.98	4.27	2.99	3.79	3.73	2.07	3.92	2.65
Loss trials	*M*	9.75	9.38	10.50	9.75	9.81	9.50	11.44	10.25
	*SD*	5.98	4.27	2.99	3.79	3.73	2.07	3.92	2.65
Total earning	*M*	20.60	27.71	28.16	30.40	28.17	30.45	23.64	28.33
	*SD*	16.90	10.91	5.46	9.99	7.32	7.58	23.35	8.38
Adjusted pumps following a win	*M*	43.77	42.10	44.38	41.77	42.71	42.74	43.00	44.57
	*SD*	22.59	12.67	10.60	7.77	8.22	3.93	13.62	7.13
Adjusted pumps following a loss	*M*	45.14	43.00	41.90	44.86	42.03	41.83	45.42	40.78
	*SD*	20.48	14.81	12.03	10.18	12.02	7.51	11.51	7.00
Reaction time (ms)	*M*	287.57	218.84	205.47	185.03	197.53	156.00	177.29	182.28
	*SD*	169.47	134.49	131.79	106.42	118.61	53.43	51.77	77.43

### fMRI experiment

#### Behavioral results

Table 2 shows the behavioral results of the fMRI experiment. A series of 2 (choice condition: passive vs. active) × 2 (time: pre- vs. post-bed) repeated-measures ANCOVAs with age as the covariate revealed that there were no significant effects of time on the total number of pumps, *F*_(1, 14)_ = 2.260, *p* = 0.155; the average number of pumps, *F*_(1, 14)_ = 1.766, *p* = 0.205; the number of wins, *F*_(1, 14)_ = 0.070, *p* = 0.795; the number of losses, *F*_(1, 14)_ = 0.142, *p* = 0.712; the total earnings, *F*_(1, 14)_ = 0.374, *p* = 0.550; the total adjusted pumps, *F*_(1, 14)_ = 0.253, *p* = 0.623; the average adjusted pumps, *F*_(1, 14)_ = 0.965, *p* = 0.343; or the reaction time, *F*_(1, 14)_ = 0.175, *p* = 0.682. A 2 (previous feedback: win or loss) × 2 (choice condition: passive vs. active) × 2 (time) repeated-measures ANCOVA with age as the covariate conducted on the average adjusted pumps following a win or a loss also revealed no significant effects of time, *F*_(1, 13)_ = 2.861, *p* = 0.115. Consistent with the results of the behavioral experiment, these results indicated that the participants' risk-taking behavior did not change with time.

**Table 2:** **The behavioral results of the fMRI experiment**.

		**Pre-bed rest test**		**Post-bed rest test**	
		**Active**	**Passive**	**Active**	**Passive**
Pumps	*M*	6.22	5.93	6.82	5.96
	*SD*	1.26	0.47	0.94	0.48
Total of pumps	*M*	101.88	95.50	108.38	100.63
	*SD*	13.55	11.21	6.63	13.14
Adjusted pumps	*M*	6.88	5.39	7.79	5.25
	*SD*	1.85	0.55	1.41	0.58
Total of adjusted pumps	*M*	63.81	59.00	63.81	58.50
	*SD*	18.97	15.08	21.42	16.16
Win trials	*M*	10.38	11.13	9.13	11.06
	*SD*	4.72	3.30	3.72	2.89
Loss trials	*M*	6.56	5.13	7.06	5.88
	*SD*	2.78	1.54	2.57	1.82
Total earning	*M*	6.84	5.39	5.97	4.08
	*SD*	7.95	8.25	11.29	8.98
Adjusted pumps	*M*	6.97	5.49	6.80	5.13
following a win	*SD*	1.94	0.63	1.70	1.19
Adjusted pumps	*M*	6.86	5.23	8.05	5.58
following a loss	*SD*	2.28	1.14	1.48	0.61
Reaction time (ms)	*M*	707.92	775.01	517.74	711.22
	*SD*	515.86	366.39	208.94	455.62

The repeated-measures ANCOVAs showed that there was no significant effect of choice condition on the average number of pumps, *F*_(1, 14)_ = 1.323, *p* = 0.269; the number of wins, *F*_(1, 14)_ = 0.211, *p* = 0.653; the number of losses, *F*_(1, 14)_ = 3.090, *p* = 0.101; the total earnings, *F*_(1, 14)_ = 0.166, *p* = 0.689; the total adjusted pumps, *F*_(1, 14)_ = 0.015, *p* = 0.904; the average adjusted pumps, *F*_(1, 14)_ = 0.616, *p* = 0.446; or the average adjusted pumps following a win or a loss, *F*_(1, 13)_ = 1.007, *p* = 0.334. However, the choice condition effect was significant for the total number of pumps, *F*_(1, 14)_ = 8.051, *p* = 0.013 and the reaction time, *F*_(1, 14)_ = 7.846, *p* = 0.014. A *post-hoc* analysis showed that the participants performed more pumps in the active choice condition than in the passive choice condition, indicating that the participants did not inflate randomly in the active choice condition. A *post-hoc* analysis also revealed that the participants' reaction time in the passive choice condition was longer than that in the active choice condition, indicating that the participants showed more active behavior in the active choice condition than in the passive choice condition.

In addition, repeated-measures ANCOVAs revealed no significant interaction effect on the total number of pumps, *F*_(1, 14)_ = 0.586, *p* = 0.457; the average number of pumps, *F*_(1, 14) = 2.096_, *p* = 0.170; the amount of wins, *F*_(1, 14)_ = 1.616, *p* = 0.224; the amount of losses, *F*_(1, 14)_ = 0.014, *p* = 0.907; the total earnings, *F*_(1, 14)_ = 0.338, *p* = 0.570; the total adjusted pumps, *F*_(1, 14)_ = 1.311, *p* = 0.271; the average adjusted pumps, *F*_(1, 14)_ = 0.362, *p* = 0.557; the average adjusted pumps following a win or a loss, *F*_(7, 98)_ = 1.396, *p* = 0.216; or the reaction time, *F*_(1, 14)_ = 1.176, *p* = 0.296. No other significant effect was found.

#### Neuroimaging results

**Figure 2:**
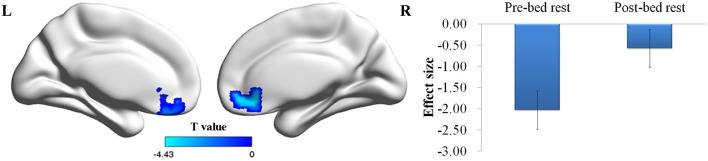
**Risk-related brain regions showing the main effect of time**. The deactivation of VMPFC decreased after the bed rest. Abbreviations: VMPFC, ventromedial prefrontal cortex. Error bar denotes the standard error.

In contrary to the behavioral results, a Two-Way ANOVA conducted on the risk-related contrasts revealed a significant main effect of time on the activation of the VMPFC. A *post-hoc t*-test found that the VMPFC showed less deactivation after the bed rest than before the bed rest (Figure 2). The main effect of the choice condition was also significant (*p* < 0.01, corrected for multiple comparisons). A *post-hoc* analysis revealed that greater activation in the active choice condition than in the passive choice condition was found in the dorsomedial prefrontal cortex, bilateral dorsolateral prefrontal cortex, bilateral insula, bilateral striatum, bilateral precentral gyrus, bilateral post-central gyrus, and left inferior parietal lobule (Table 3). No interaction effect was found.

**Table 3:** **The results for risk-related brain regions identified in the *post-hoc t*-tests**.

**Region**	**Cluster size**	**BA**	**Peak *t*-values**	**Peak MNI coordinates**		
**MAIN EFFECT OF TIME: BEFORE < AFTER**						
Ventromedial prefrontal cortex	146	11	3.25	12	48	−6
**MAIN EFFECT OF CHOICE CONDITION: ACTIVE > PASSIVE**						
Bilateral insula/striatum/precentral gyrus/post-central gyrus/dorsomedial prefrontal cortex/dorsolateral prefrontal cortex	11451	3/6/7/9/10/13/24/32/40/44/47	7.65	9	9	48
Left inferior parietal lobule	394	7/40	5.58	−36	−51	45
**INTERACTION BETWEEN TIME AND CHOICE CONDITION**						
None						

**Figure 3:**
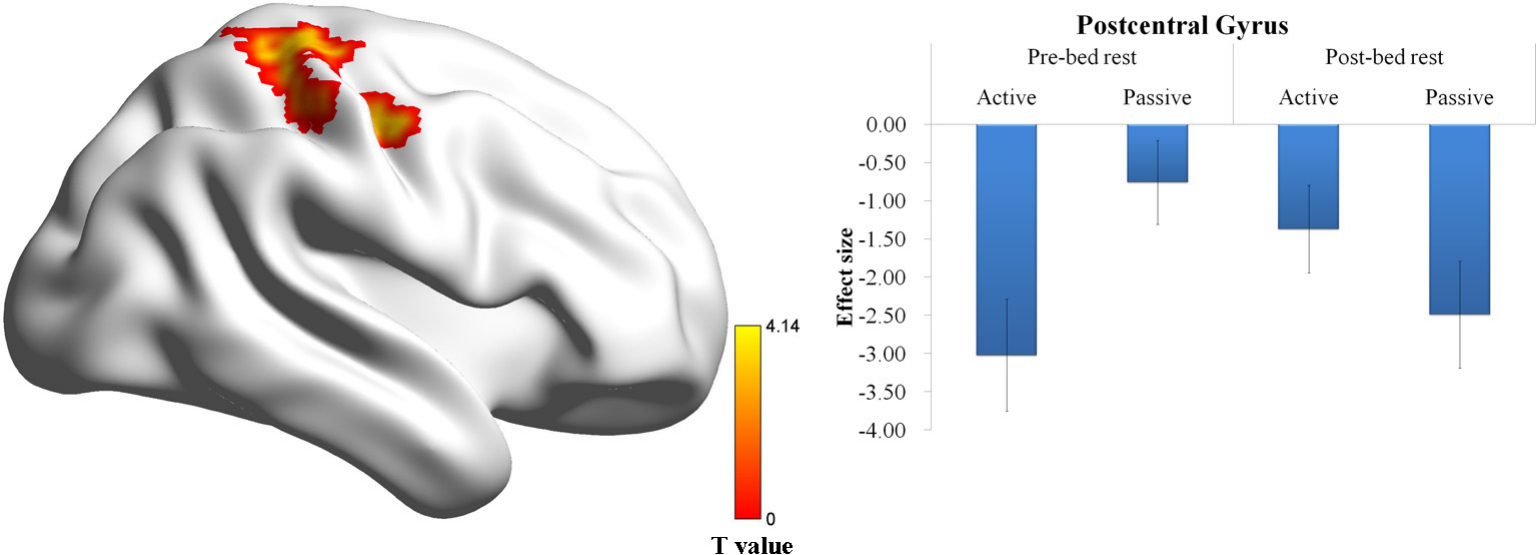
**Outcome-related brain regions showing the Two-Way interaction between time and choice condition**. The activation of post-central gyrus in the active choice condition was greater after the bed rest. No significant difference between before and after bed rest was found in the passive choice condition. Error bar denotes the standard error.

**Figure 4:**
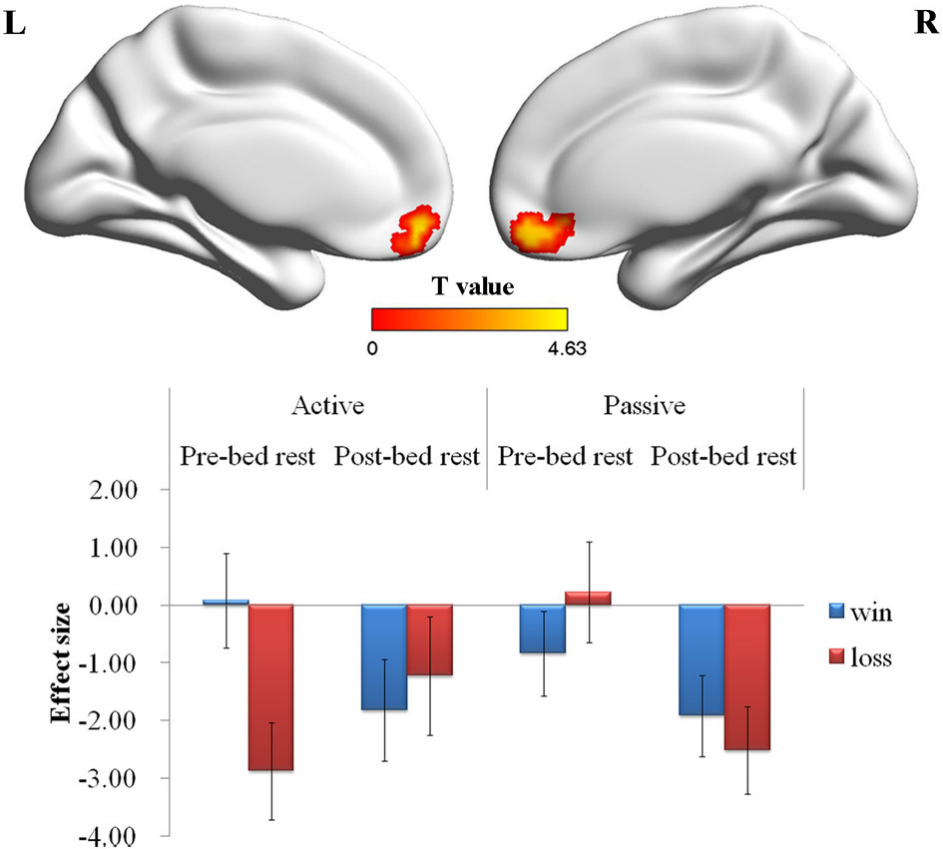
**Outcome-related brain regions showing the Three-Way interaction**. Before bed rest, the activation of VMPFC in the active choice condition was greater in the win outcome than in the loss outcome, whereas it showed no significant difference between the win and loss outcomes after bed rest. In contrast, the activation of VMPFC in the passive choice condition did not differ between win and loss outcomes, either before bed rest or after bed rest. Abbreviations: VMPFC, ventromedial prefrontal cortex. Error bar denotes the standard error.

A Three-Way ANOVA conducted on the outcome-related contrasts revealed a significant Two-Way interaction on the activation of the post-central gyrus (*p* < 0.01, corrected for multiple comparisons) and a significant Three-Way interaction on the activation of the VMPFC (*p* < 0.01, corrected for multiple comparisons). *Post-hoc* analysis on the Two-Way interaction revealed that the activation of post-central gyrus in the active choice condition was greater after the bed rest (Figure 3). *Post-hoc* analysis on the Three-Way interaction revealed that the activation of VMPFC in the active choice condition was significantly greater in the win outcome than in the loss outcome before bed rest, whereas it showed no significant difference between win and loss outcomes after bed rest. In contrast, the activation of VMPFC in the passive choice condition did not differ between win and loss outcomes, either before bed rest or after bed rest (Figure 4).

In addition, the Three-Way ANOVA revealed several effects unrelated to time. The main effect of outcome was significant on the activation of the dorsomedial prefrontal cortex, bilateral inferior frontal gyrus/insula, left cerebellum posterior lobe, and right post-central gyrus (Table 4) (*p* < 0.01, corrected for multiple comparisons). Further analysis revealed that the activation of the left cerebellum posterior lobe and right post-central gyrus was greater in the win outcome than in the loss outcome, whereas the activation of the dorsomedial prefrontal cortex and bilateral inferior frontal gyrus/insula showed the opposite pattern. The activation of the striatum was greater in the passive choice condition than in the active choice condition (Table 4) (*p* < 0.01, corrected for multiple comparisons). The interaction between outcome and choice condition was significant for the activation of a large cluster including the bilateral precuneus, lingual gyrus, posterior cingulate cortex, cerebellum posterior and anterior lobe (Table 4) (*p* < 0.01, corrected for multiple comparisons). Further analysis revealed that these regions showed a greater activation in the win outcome than in the loss outcome in the active choice condition, whereas the opposite pattern was observed in the passive choice condition.

**Table 4:** **The result of outcome-related brain regions identified in the *post-hoc t*-tests**.

**Region**	**Cluster size**	**BA**	**Peak *t*-values**	**Peak MNI coordinates**		
**MAIN EFFECT OF TIME**						
None						
**MAIN EFFECT OF CHOICE CONDITION: ACTIVE < PASSIVE**						
Striatum	77		4.43	−6	9	6
**MAIN EFFECT OF OUTCOME: WIN > LOSE**						
Left cerebellum posterior lobe	193		5.82	−24	−54	−54
Right post-central gyrus	525	1/2/3/4/6	5.29	27	−30	75
**MAIN EFFECT OF OUTCOME: WIN < LOSE**						
dorsomedial prefrontal cortex	408	6/8/9	3.17	−3	27	39
Left inferior frontal gyrus/insula	461	13/47	3.17	−51	27	3
Right inferior frontal gyrus/insula	319	13/47	3.19	42	18	−18
**INTERACTION BETWEEN TIME AND CHOICE CONDITION**						
Right post-central gyrus	152	3/4/5	4.14	36	−30	51
**INTERACTION BETWEEN TIME AND OUTCOME**						
None						
**INTERACTION BETWEEN CHOICE CONDITION AND OUTCOME**						
Bilateral precuneus/lingual	3460	7/17/	6.35	6	−90	−18
gyrus/posterior cingulate		18/19/				
cortex/cerebellum posterior		23/24/				
lobe/cerebellum anterior lobe		31/37				
**THREE-WAY INTERACTION**						
Ventromedial prefrontal cortex	96	11	4.63	−3	54	−15

## Discussion

In the present study, we used the BART to investigate the effect of bed rest on individuals' risk-taking behavior. Following Lejuez et al. (2002), the average number of pumps only on trials when the participant cashed out before the balloon exploded provided a measure of the risk-taking behavior threshold for individual participants. The analysis of this measure, as well as other indexes obtained in the BART, showed that all of these measures were not affected by bed rest. This is possibly because the behavioral test might not be sensitive enough to detect the effect of bed rest on risk-taking.

In contrast to the behavioral results, the neuroimaging results showed that the VMPFC showed less deactivation subsequent to bed rest. In the field of neuroeconomics, the VMPFC has been implicated as a principal component of decision-making circuitry during risky decision making (Bechara et al., 1999; Kable and Glimcher, 2009; Xue et al., 2009). Specifically, the VMPFC was reported to be involved in value calculation (Rushworth et al., 2011). Our finding that VMPFC activation in the active choice condition showed significant differences between the win and loss outcomes before bed rest might provide further evidence to support a role of value calculation for the VMPFC. Consistent with a previous study (Schonberg et al., 2012), we found that VMPFC activation decreased as the participants further expanded the balloons. Previous researchers had suggested that the decreasing VMPFC activation implied that escalating risk taking in the BART might be perceived as exposure to increasing possible losses rather than the increasing potential total reward relative to the starting point (Schonberg et al., 2012). Given this consideration, the significant decrease in VMPFC deactivation after bed rest suggests that the participants showed a decreased level of value calculation after bed rest. This interpretation was further supported by our finding that the VMPFC activation between win and loss outcomes did not significantly differ after bed rest. In short, our finding might imply a detrimental effect of bed rest on risky decision making. However, this explanation is tentative and warrants further investigation.

In addition, the finding that VMPFC showed less deactivation subsequent to bed rest suggested that the cerebral cortex plasticity could change after bed rest. A previous study has shown evidence of overall decreased leg corticospinal excitability during prolonged bed rest (Roberts et al., 2010). This possibility is also supported by our finding that the activity of the post-central gyrus in the active choice condition during the outcome presentation changed after bed rest. The blood oxygenation level dependent response can be characterized by the hemodynamic response function, and different hemodynamic response functions show various levels of statistical sensitivity for detecting an evoked activity in different brain regions (Badillo et al., 2013). Therefore, future research might examine whether hemodynamic response functions change after bed rest.

Risk taking refers to an active willingness to pursue an opportunity. Previous studies have found that risk shows robust activation in the medial and dorsalateral prefrontal cortex, insula, striatum, and midbrain in the active choice condition rather than in the passive choice condition (Rao et al., 2008; Cazzell et al., 2012; Lighthall et al., 2012; Telzer et al., 2013). Consistent with previous studies, we found greater activation in the active than in the passive choice condition in the dorsomedial prefrontal cortex, bilateral insula, striatum, dorsalateral prefrontal cortex, and left inferior parietal lobule. This similarity implies that our analyses and results are reliable. The frontal-parietal network plays an important role in organizing goal-directed behaviors (Barash, 2003). Specifically, the inferior parietal lobule is involved in the integration of information about probabilities and gain/loss magnitudes (Labudda et al., 2008). The greater activation in the frontal-parietal network in the active choice condition than in the passive one suggests that the participants conducted a deliberative evaluation, including integrating information about risk and rewards, when they made an active decision. Such findings provide a useful insight into the important role of active willingness in risk taking.

Several issues raised by this study merit consideration. First, the prolonged period of bed rest and lack of social contacts may cause an increase in stress responses. A previous study has found that stress influences the risk-taking behavior in the BART (Lighthall et al., 2009). However, the finding that the risk-taking behavior remains unchanged after the bed rest implies that the stress effect may be weak to be detected. Future studies should include more psychological measures, such as stress, to investigate whether stress may influence risk-taking behaviors in a bed rest study. Second, note that gender differences have been found in risk-taking behavior (Hallahan et al., 2004; Lighthall et al., 2009; Cazzell et al., 2012). Our results are limited to males because only males were included in the present study. As women have been found to demonstrate less tolerance for risk and more risk aversion than males in the willingness to take financial risks (Hallahan et al., 2004), future studies should consider incorporating females as participants in the bed rest study. Third, a limitation of our study might be that we did not include an ambulatory control group. However, the passive choice condition in the fMRI experiment would help to overcome this limitation, as the passive choice condition could serve as a control condition compared with the active choice condition. In addition, we controlled for body posture across all phases of testing, including pre-bed rest, during bed rest, and post-bed rest. Finally, certain researchers have noted that several improvement effects on executive functioning during bed rest might be due to practice (Lipnicki and Gunga, 2009). In the present study, there was no significant effect of time on reaction time or on the participants' performance, indicating that there was no significant practice effect.

Because bed rest simulates microgravity, the results of bed rest could be important for astronauts on long-duration spaceflights. However, because bed rest cannot be equated with the conditions that astronauts experience during space flights, any effects of bed rest may underestimate the true size of the potential effects. The investigation of risk-taking in bed rest studies is nevertheless considered to contribute to a more appropriate selection and training of astronauts. Moreover, the results of bed rest studies might shed light on the understanding of the cognitive impairments associated with medical conditions involving restricted physical activity, as well as the impairments that affect individuals with a sedentary lifestyle (Lipnicki and Gunga, 2009).

### Conflict of interest statement

The authors declare that the research was conducted in the absence of any commercial or financial relationships that could be construed as a potential conflict of interest.
